# Potassium as a pluripotency-associated element identified through inorganic element profiling in human pluripotent stem cells

**DOI:** 10.1038/s41598-017-05117-2

**Published:** 2017-07-10

**Authors:** Victor J. T. Lin, Ashwini Zolekar, Yi Shi, Bhuvaneswari Koneru, Slobodan Dimitrijevich, Anthony J. Di Pasqua, Yu-Chieh Wang

**Affiliations:** 1 Department of Pharmaceutical Sciences, University of North Texas Health Science Center, Fort Worth, TX USA; 2 Cardiovascular Research Institute, University of North Texas Health Science Center, Fort Worth, TX USA; 3 UHV Technologies, Inc., Fort Worth, TX USA; 4 Department of Pharmaceutical Sciences, School of Pharmacy, Binghamton University, Binghamton, NY USA

## Abstract

Despite their well-known function in maintaining normal cell physiology, how inorganic elements are relevant to cellular pluripotency and differentiation in human pluripotent stem cells (hPSCs) has yet to be systematically explored. Using total reflection X-ray fluorescence (TXRF) spectrometry and inductively coupled plasma mass spectrometry (ICP-MS), we analyzed the inorganic components of human cells with isogenic backgrounds in distinct states of cellular pluripotency. The elemental profiles revealed that the potassium content of human cells significantly differs when their cellular pluripotency changes. Pharmacological treatment that alters cell membrane permeability to potassium affected the maintenance and establishment of cellular pluripotency via multiple mechanisms in *bona fide* hPSCs and reprogrammed cells. Collectively, we report that potassium is a pluripotency-associated inorganic element in human cells and provide novel insights into the manipulation of cellular pluripotency in hPSCs by regulating intracellular potassium.

## Introduction

Human pluripotent stem cells (hPSCs), including human embryonic stem cells (hESCs) and induced pluripotent stem cells (hiPSCs), are capable of differentiation into all types of somatic cells in the human body^1^. During differentiation, hPSCs lose their cellular pluripotency and commit themselves to specific lineages. Since the transition between pluripotent and non-pluripotent states in hPSCs is orchestrated by highly dynamic and intricate signaling networks^1^, factors that broadly influence cell signaling are almost certain to have an impact on the regulation of cellular states in hPSCs. Indeed, a substantial amount of molecular features and regulatory mechanisms relevant to cellular pluripotency at the transcriptomic^2–4^, epigenetic^2, 5, 6^, protein expression^7, 8^, post-translational modification^1, 7, 9–12^, and metabolomic^13, 14^ levels have been discovered. Although the knowledge about hPSCs and their utility has rapidly expanded in the past decade, the regulatory mechanism of cellular pluripotency is still not fully understood.

Many inorganic elements, including the most recently identified essential element, bromine^15^, are extensively involved in the modulation of biochemical reactions and cell signaling pathways^16–19^. The abnormal distributions of inorganic elements are often observed in different types of diseased cells^20–23^, highlighting the important role that inorganic elements play in the regulation of cellular states and normality. In contrast, normal cells in different cell lineages and physiological conditions can express or store certain metalloproteins (*e.g*., metallothionein, ferritin, superoxide dismutase, metalloprotease, and hemoglobin), coenzymes (*e.g*., vitamin B12) and hormones (*e.g*., thyroxine) that contain inorganic cofactors^16^. This suggests that human cells in unique states, such as undifferentiated hPSCs and their differentiated derivatives, are likely to have specific requirements for different inorganic elements and display characteristic elemental profiles. Using synchrotron X-ray fluorescence spectroscopy, the polarized distribution of specific elements was observed in neurospheres differentiated from mouse embryonic stem cells (mESCs) and WA09 hESCs^24^. A recent study also revealed that zinc (Zn) appears to play an important role in the maintenance of cellular pluripotency in mESCs^25^. However, the significance of inorganic elements for hPSCs in pluripotent and non-pluripotent states has not been systematically investigated.

Here, we use total reflection X-ray fluorescence (TXRF) spectrometry and inductively coupled plasma mass spectrometry (ICP-MS) techniques to characterize the content of inorganic elements in a panel of undifferentiated hPSCs, their isogenic differentiated derivatives, and somatic cells used for reprogramming. Our results show that the intracellular content of potassium differs considerably between pluripotent and non-pluripotent cells. The perturbation of potassium homeostasis affects the core components of pluripotency signaling in *bona fide* hPSCs and influences the efficiency of cell reprogramming for hiPSC production. The mechanisms that are potentially involved in the intracellular potassium-associated alteration of cellular pluripotency and its possible application were also examined.

## Results

### Intracellular potassium content differs between hPSCs and non-pluripotent counterparts

**Figure 1:**
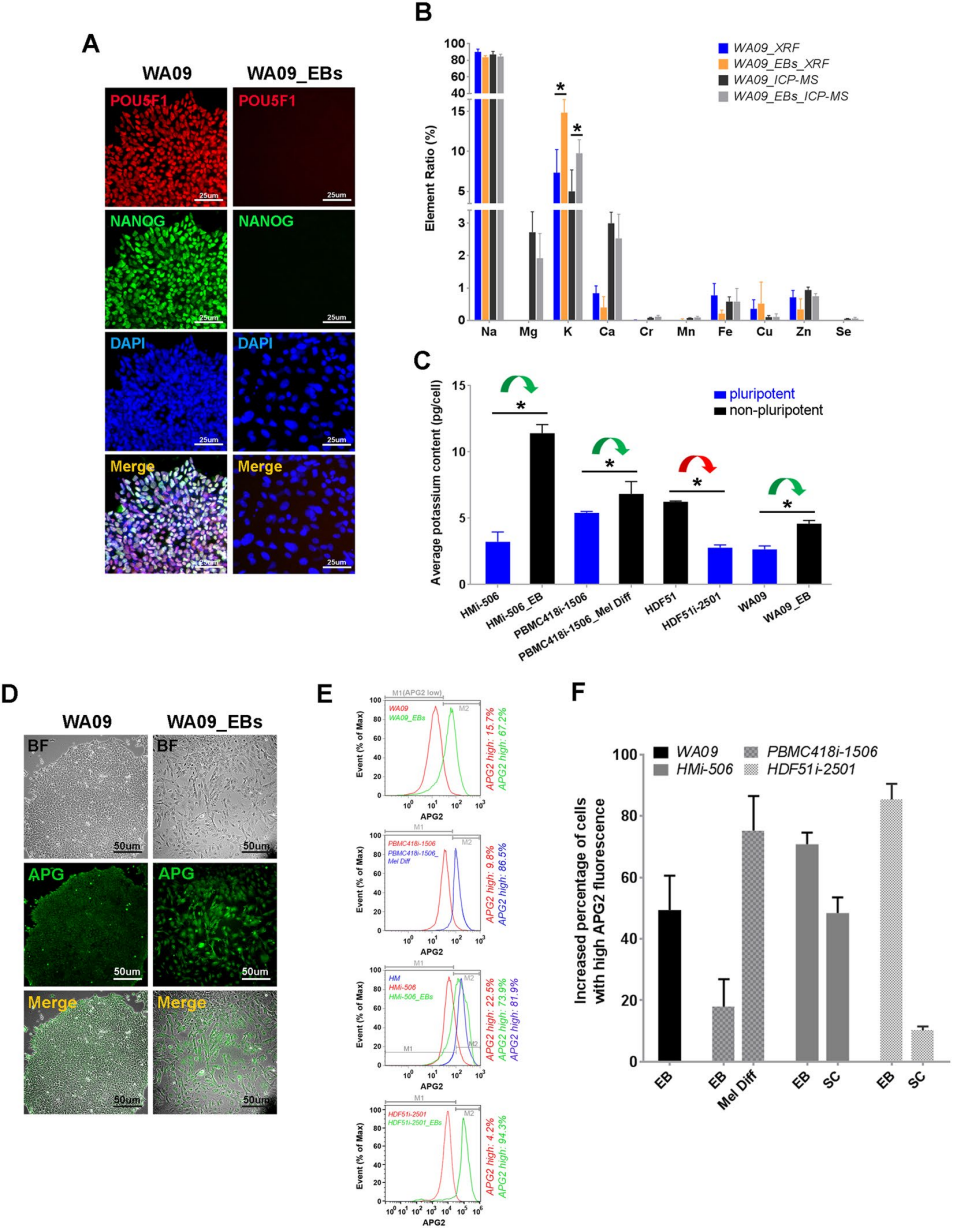
Intracellular potassium content differs in human pluripotent and non-pluripotent cells. (**A**) Staining of POU5F1 and NANOG in undifferentiated WA09 hESCs and their differentiated derivatives (WA09_EBs). (**B**) TXRF and ICP-MS profiling of 10 inorganic elements showed WA09 hESCs contained lower intracellular potassium compared to WA09_EBs. (**C**) ICP-MS analysis showed that undifferentiated hiPSCs (including HMi-506, PBMC418i-1506, and HDF51i-2501 cells) generally contained lower intracellular potassium compared to their isogenic non-pluripotent cells (including HMi-506_EB, PBMC418i-1506_Mel Diff, and HDF51 cells). *Green arrow:* differentiation. *Red arrow:* Sendai virus-mediated cell reprogramming for hiPSC derivation. (**D**) APG2 staining in WA09 hESCs and WA09_EBs. (**E**) Representative histograms of APG2-stained cells from flow cytometry analysis. *Top panel:* WA09 hESCs and WA09_EBs. *First middle panel:* PBMC418i-1506 hiPSCs and their melanocytic derivatives (PBMC418i-1506_Mel Diff). *Second middle panel:* HMi-506 hiPSCs and isogenic non-pluripotent cells (HM and HMi-506_EBs). *Bottom panel:* HDF51i-2501 hiPSCs and HDF51i-2501_EBs. (**F**) Increased percentages of cells with high APG2 fluorescence in 7 isogenic pairs of pluripotent and non-pluripotent samples (*EB:* embryoid bodies of the indicated hPSCs, *Mel Diff:* melanocytic derivatives of the indicated hPSCs, *SC:* somatic cells used for generating hiPSCs). All data are presented as mean ± standard deviation (*n* = 3; *P < 0.05, *t*-test) in each bar graph.

To address the significance of inorganic components in hPSCs and non-pluripotent cells, we profiled the relative content of 56 inorganic elements using TXRF spectrometry in undifferentiated WA09 hESCs and their differentiated derivatives obtained from embryoid body (EB) formation. The loss of cellular pluripotency in the differentiated derivatives (WA09 EBs) was confirmed by the major reduction of POU5F1 and NANOG expression (Fig. 1A). In the initial profiling, a few inorganic elements including sodium (Na), phosphorus (P), sulfur (S), chloride (Cl) and potassium (K) appeared to be highly abundant in both undifferentiated and differentiated WA09 hESCs (Figure S1). The abundance of these elements was expected since they are either the structural components of biological macromolecules or the pivotal mediators of osmolarity and cell membrane potential. In addition to the abundant elements, several trace and ultra-trace elements, including calcium (Ca), iron (Fe), copper (Cu), manganese (Mn) and Zn, known for their essential roles in keeping normal cell function^16, 17^ were also detectable in the undifferentiated and differentiated cells. Our data suggest that TXRF analysis could be used to detect and compare the contents of multiple inorganic elements across different hPSC samples.

To validate the TXRF results and identify pluripotency-associated inorganic elements, we measured 10 elements of interest using ICP-MS analysis in the same set of cell samples (3 from undifferentiated WA09 hESCs and 3 from WA09 EBs) that were analyzed using the TXRF technique. The profiles of these 10 elements determined by TXRF and ICP-MS analyses were largely similar, with few exceptions like magnesium (Mg) and Ca (Fig. 1B). Comparing the relative contents of the selected elements in WA09 hESCs and WA09 EBs, WA09 EB cells contained a significantly higher amount of potassium, indicated by both TXRF and ICP-MS analyses (Fig. 1B). Using ICP-MS analysis, we further tested potassium contents in a set of hiPSCs, their differentiated derivatives, and somatic cells used for reprogramming. Despite the different somatic cell origins and distinct reprogramming methods (Table S1), non-pluripotent cells showed significantly higher potassium contents in every isogenic pair of hiPSCs and non-pluripotent cells that we analyzed (Fig. 1C). The difference in potassium between hPSCs and their isogenic non-pluripotent cells was also revealed at the cellular level using a cell-permeable fluorescence indicator for potassium, APG2-AM. Compared with hPSCs, their isogenic non-pluripotent cells generally had an increased percentage of cells with high APG2 fluorescence (Fig. 1D–F), indicating the higher amount of potassium in non-pluripotent cells. Using ICP-MS, we also tested potassium contents of the media for culturing undifferentiated hPSCs and for generating their EBs. The potassium content of the medium for culturing hPSCs was ~203 ppm, whereas the medium for generating EBs contained ~165 ppm of potassium. These results suggest that the potassium content of human cells differs when their cellular pluripotency changes, independent of potassium contents in the culture media. In addition, potassium is a pluripotency-associated inorganic element.

### Potassium channel modulators and a potassium ionophore affect the core components of pluripotency signaling in hPSCs

**Figure 2:**
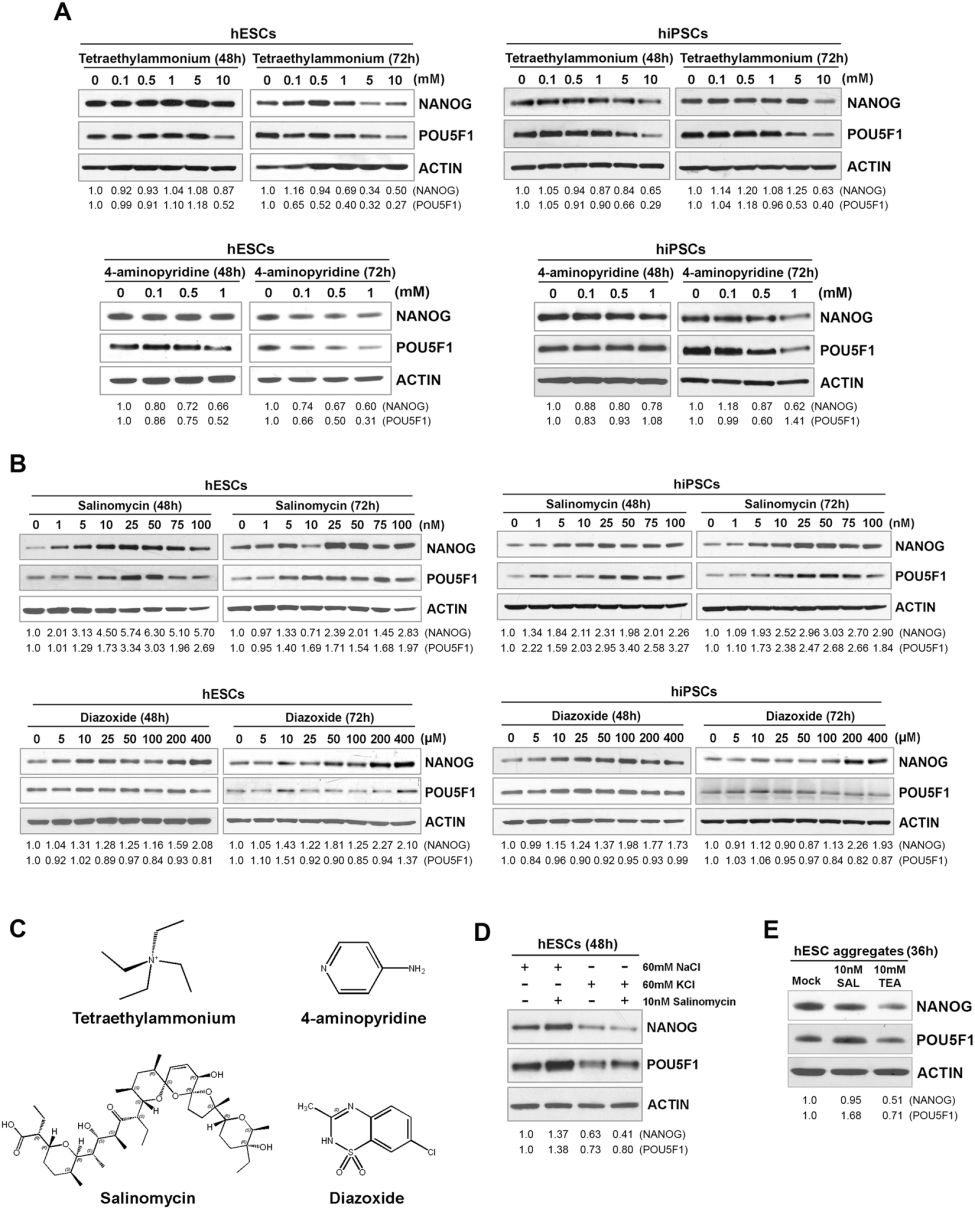
Alteration in NANOG and POU5F1 protein expression in WA09 hESCs and HDF51i-509 hiPSCs treated with potassium channel modulators and a potassium ionophore. (**A**) Potassium channel blockers (tetraethylammonium and 4-aminopyridine) induced the dose- and time-dependent downregulation of NANOG and POU5F1 detected by Western blotting. (**B**) A potassium ionophore, salinomycin, caused a dose- and time-dependent upregulation of NANOG and POU5F1. NANOG was upregulated by a potassium channel activator, diazoxide, in both hESCs and hiPSCs, while POU5F1 appeared to be relatively unaffected. (**C**) The chemical structures of the potassium channel modulators and ionophore. (**D**) The expression and salinomycin-induced upregulation of NANOG and POU5F1 in WA09 hESCs were attenuated by adding 60 mM KCl in the culture medium. (**E**) The 36-hour treatment of TEA promotes the downregulation of NANOG and POU5F1 in WA09 hESC aggregates cultured in the FGF-deficient medium at the beginning of EB formation. Normalized NANOG and POU5F1 protein band intensity from densitometry analysis was shown at the bottom of each panel of Western blotting images. Representative blot images displayed in this figure were cropped from original blots shown in the Supplementary Information and organized into composite panels.

We next tested the effect of small molecules that alter cell membrane permeability to potassium in hPSCs. Consistent with the previous finding in mESCs^26^, tetraethylammonium (TEA) and 4-aminopyridine (4-AP) that block voltage-gated potassium channels and suppress the efflux of intracellular potassium^27^ increased intracellular potassium (Figure S2A) and caused the dose- and time-dependent downregulation of POU5F1 and NANOG in hESCs and hiPSCs (Fig. 2A). Since the TEA-treated WA09 hESCs barely showed increase of apoptosis (Figure S2B), the downregulation of pluripotency factors in the TEA-treated cells was not due to cytotoxicity and cell death. In contrast, potassium channel activator diazoxide (DZ) and ionophore salinomycin (SAL), which promote the efflux of intracellular potassium^28–30^, led to the reduction of intracellular potassium (Figure S2A) and the upregulation of POU5F1 and NANOG in hESCs and hiPSCs (Fig. 2B). These results indicate that the modulators of membrane permeability to potassium can bias cellular pluripotency in hPSCs. Due to the distinct structures of these small molecules (Fig. 2C), it seems unlikely that the similar effects of TEA and 4-AP, as well as the similar effects of DZ and SAL on different hPSCs, were linked to the off-target effects of each small molecule rather than their common effect in altering intracellular potassium.

Compared with the medium that was spiked with sodium chloride, the potassium chloride-spiked medium greatly attenuated the expression and SAL-induced upregulation of NANOG and POU5F1 in hESCs (Fig. 2D). Since the addition of potassium chloride in the medium counteracts the normal potassium gradient across the cell membrane, the response of hESCs in the potassium chloride-spiked medium is possibly due to the diminished efflux and elevated concentration of intracellular potassium. Interestingly, TEA treatment also promoted the downregulation of POU5F1 and NANOG in WA09 hESCs that underwent differentiation and EB formation (Fig. 2E), indicating that an increase in intracellular potassium content may propel hPSCs to exit their pluripotent state during differentiation. These findings further support our hypothesis that intracellular potassium is not only correlated with the pluripotent state of hPSCs but also involved in the regulation of their cellular pluripotency.

**Figure 3:**
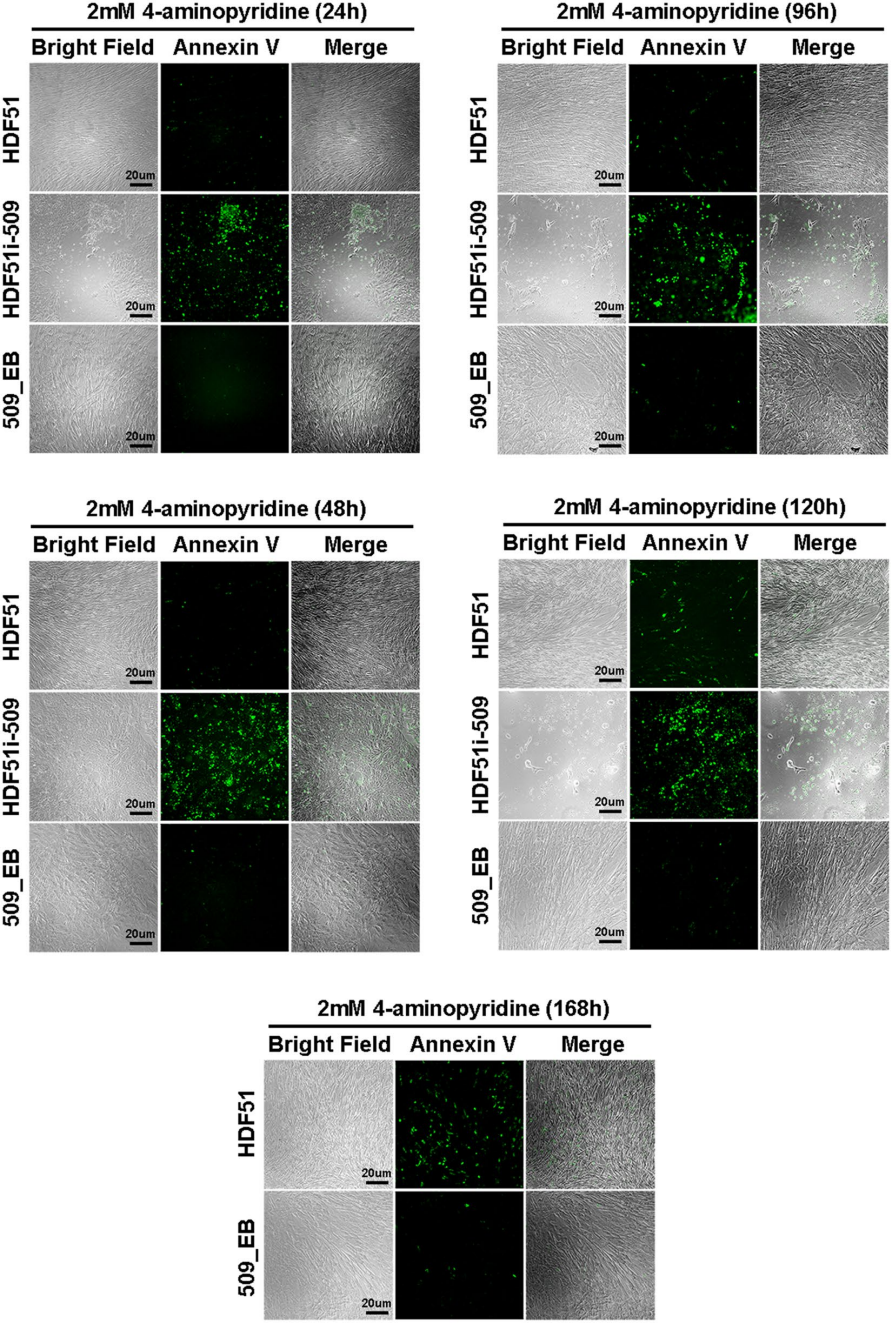
Potassium channel blocker 4-aminopyridine at 2 mM preferentially induced apoptosis in undifferentiated hPSCs in a time-dependent manner. Staining of Alexa Fluor 488-conjugated annexin V in hiPSCs (HDF51i-509), their differentiated derivatives (509_EB) and somatic cells used for reprogramming (HDF51) with the treatment of 2 mM 4-aminopyridine indicated that most hiPSCs were eliminated by the treatment within 96 hours due to apoptosis. In contrast, non-pluripotent cells (HDF51 and 509_EB) showed limited cytotoxicity in response to the treatment.

In addition to the effect of low-dose (≤1 mM) 4-AP on pluripotency signaling, apoptosis was preferentially induced by this potassium channel blocker at higher concentrations in hPSCs, compared with their isogenic non-pluripotent cells (Fig. 3 and Figure S3). This differential cytotoxic response indicates the high susceptibility of hPSCs to 4-AP and the potential use of 4-AP as a selective agent for eliminating residual pluripotent cells from hPSC-differentiated derivatives.

### The effect of a potassium channel blocker and a potassium ionophore on cell reprogramming

**Figure 4:**
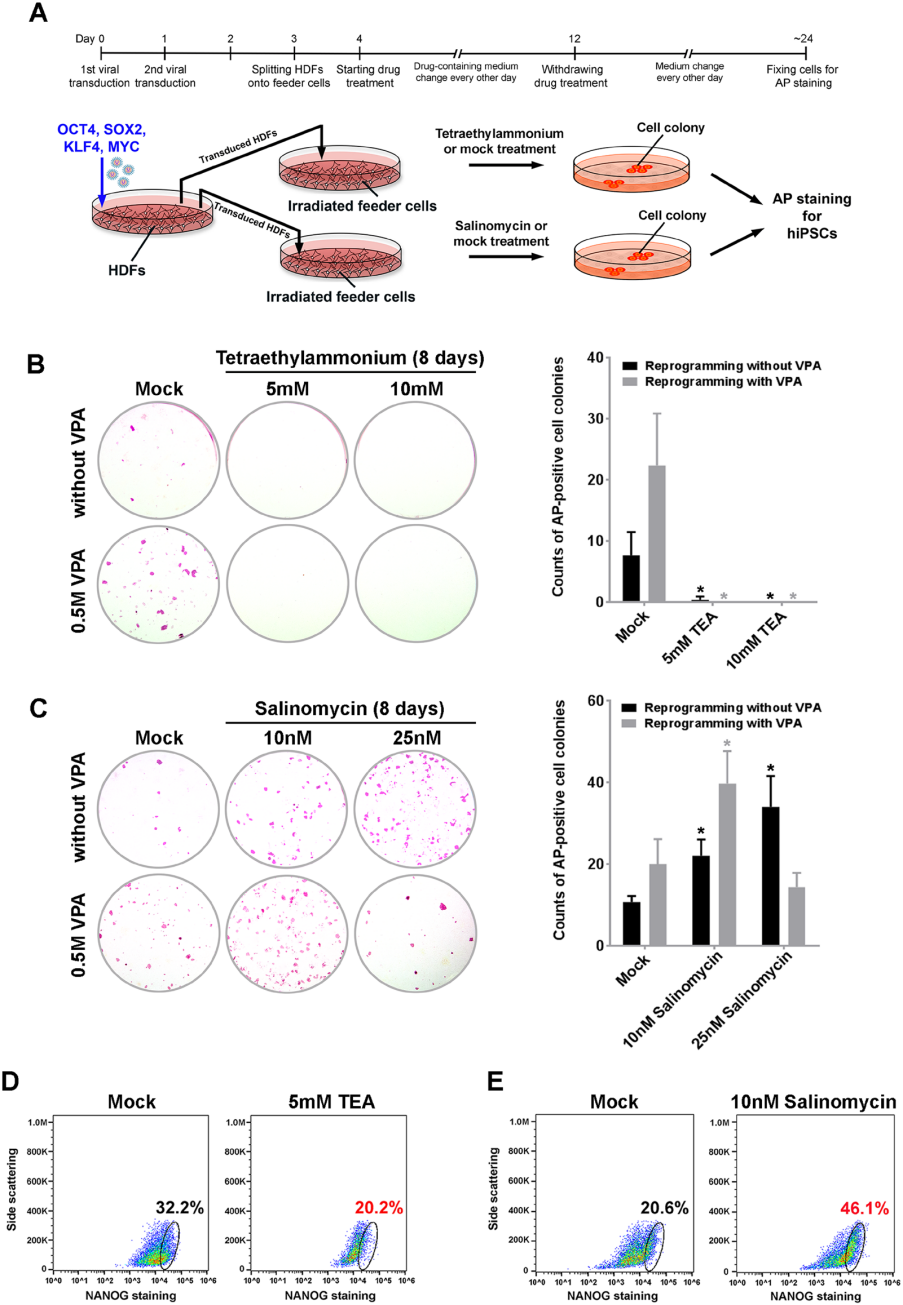
The influence of a potassium channel blocker and ionophore on the establishment of induced cellular pluripotency. (**A**) The schematic illustration of testing potassium permeability modulators on reprogrammed cells. (**B**) 5 and 10 mM tetraethylammonium (TEA) abolished hiPSC formation during cell reprogramming with and without valproic acid (VPA). *Left panel:* representative images of AP-positive cell colonies. *Right panel:* counts of AP-positive cell colonies. (**C**) 10 nM salinomycin (SAL) enhanced hiPSC formation with and without VPA. 25 nM SAL enhanced hiPSC formation without VPA but appeared to reduce hiPSC formation in the presence of VPA. *Left panel:* representative images of AP-positive cell colonies. *Right panel:* counts of AP-positive cell colonies. (**D**) Flow cytometry analysis showed that 5 mM TEA reduced NANOG-expressing cells in VPA assisted reprogramming. (**E**) 10 nM SAL increased the amount of NANOG-expressing cells induced by cell reprogramming without VPA. Data in (**D**) and (**E**) were obtained from samples collected at 14 days after the initial transduction. All data are presented as mean ± standard deviation (*n* = 3; *P < 0.05, *t*-test) in each bar graph.

Having demonstrated that the chemical-induced alteration of cell membrane permeability to potassium perturbs the core components of pluripotency signaling in *bona fide* hPSCs, we wanted to test whether the small molecules that reduce and augment the efflux of intracellular potassium influence cell reprogramming and the establishment of induced cellular pluripotency in somatic cells. Cell reprogramming in human dermal fibroblasts (HDFs) was performed using four transcription factors with and without TEA and SAL treatment (Fig. 4A). Reprogramming efficiency was assessed by alkaline phosphatase (AP) staining to detect hiPSC colonies and by flow cytometric analysis of NANOG expression. The treatment of 5 mM and 10 mM TEA for 8 days at the early stage of cell reprogramming abolished the formation of hiPSCs induced by the ectopic expression of POU5F1, SOX2, KLF4, and MYC in HDFs (Fig. 4B), independent of the presence of valproic acid (VPA) during the reprogramming. The expression of NANOG in reprogrammed cells with VPA treatment was also reduced by TEA (Fig. 4D). Since VPA is known as one of potent enhancers to facilitate the induction of pluripotency during cell reprogramming by promoting expression of pluripotency-specific genes and dowregulating differentiated cell-specific genes^31^, our results suggest that the increase of intracellular potassium may strongly bias the gene expression in the reprogrammed cells towards an expression profile specific for differentiated cells, unable to be overturned by VPA treatment. The 7-day treatment of TEA or SAL resulted in the negligible reduction of viability in mouse embryonic fibroblasts (MEFs) that were used as feeder cells to support reprogrammed HDFs (Figure S4). Thus, the TEA-induced reduction in reprogramming efficiency should primarily be attributed to the direct effect of this potassium channel blocker on reprogrammed cells rather than the treatment-caused deficiency of feeder cells.

While 10 nM SAL promoted hiPSC formation and NANOG expression in HDFs reprogrammed with and without VPA (Fig. 4C and E), 25 nM SAL appeared to impede the VPA-assisted reprogramming of HDFs (Fig. 4C). These findings attest to not only the significance of cell membrane permeability to potassium in the regulation of cell reprogramming but also the intricacy of modulating intracellular potassium to facilitate the establishment of induced pluripotency. Although higher doses of SAL were used in other studies, it has been shown that SAL at certain concentrations can synergize with different anticancer agents including HDAC inhibitors to trigger cytotoxic responses in multiple types of transformed cells^32–34^. This further suggests that the promotive and suppressive effects of SAL on reprogrammed cells could be context-dependent and varied according to its dose and treatment period.

### The alteration of cell membrane permeability to potassium affects cell reprogramming and cellular pluripotency through multiple mechanisms

**Figure 5:**
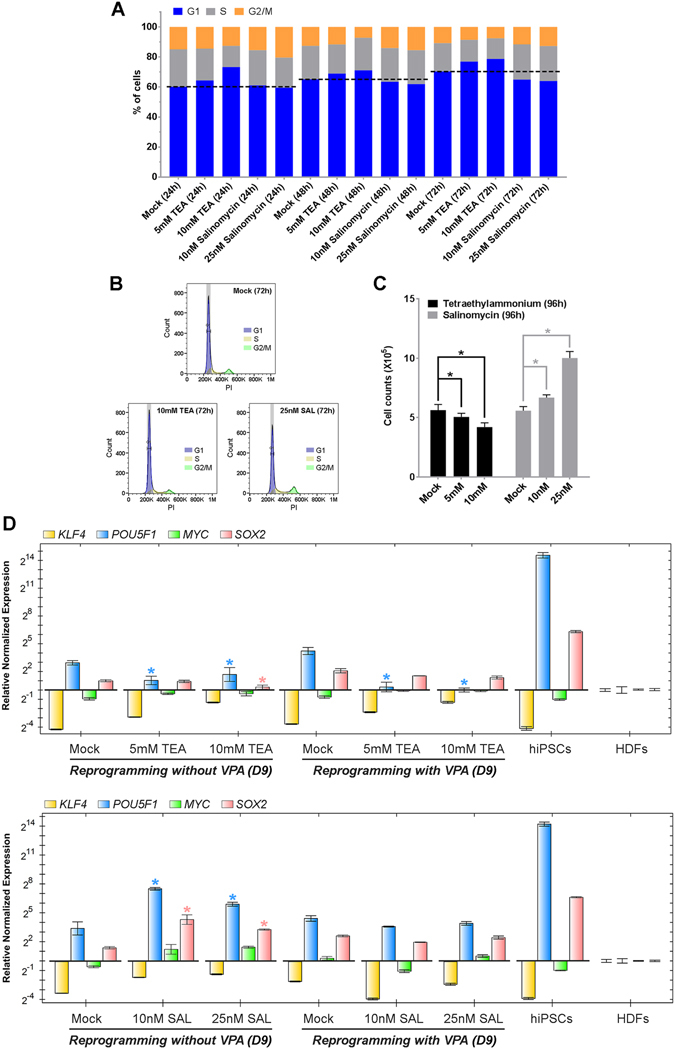
The effects of a potassium channel blocker and ionophore on cell cycle, proliferation and endogenous gene expression in HDFs used for cell reprogramming. (**A**) Cell cycle analysis indicated that tetraethylammonium (TEA) moderately prolonged the G1 phase in a dose-dependent manner. In contrast, salinomycin (SAL) appeared to facilitate G1 phase progression. (**B**) Histograms representing cellular DNA content revealed that the 72-hr treatment of 10 mM TEA increased the number of cells in the G1 phase (G1 cells), while the 72-hr treatment of 25 nM SAL decreased the number of G1 cells. Neither TEA nor SAL caused noticeable cell death, supported by the absence of an increase in sub-G1 cells. (**C**) The number of HDFs (1 × 10^5^) after the indicated 96-hr treatment was determined by cell counting (*P < 0.05, *t*-test). (**D**) The expression of the endogenous *POU5F1*, *SOX2*, *KLF4* and *MYC* genes in HDFs that underwent reprogramming with TEA and SAL was measured by qRT-PCR. Early in reprogramming, the induction of endogenous *POU5F1* and *SOX2* gene expression with and without VPA was suppressed by TEA (*upper panel*), but increased by SAL without VPA (*lower panel*). The induction of both genes was relatively unchanged by SAL in reprogramming with VPA (*P < 0.05, *t*-test, fold change ≥2). *D9*: cell samples collected at 9 days after the initial transduction (5 days after treatments began). All data are presented as mean ± standard deviation (*n* = 3) in each bar graph.

To further understand how intracellular potassium changes may affect cell reprogramming, we first tested the influence of TEA and SAL on cell cycle progression in the somatic cells used for reprogramming. Since cell membrane permeability to potassium oscillates with cell cycle progression^35^, it is believable that the perturbation of its permeability may influence cell cycle and cell proliferation. As expected, TEA prolonged the G1 phase of the cell cycle and reduced proliferation in HDFs used for cell reprogramming (Fig. 5A–C). SAL appeared to alleviate G1 cell cycle arrest that was caused by increased cell density and contact inhibition^36^, allowing them to stay active in proliferation during 96 hours of culturing (Fig. 5A–C). Similar to our findings, growth inhibition induced by potassium channel blockers was observed in WA01 (H1) hESCs, hiPSCs and other cell types^37–39^. Moreover, cell membrane hyperpolarization caused by increased potassium permeability was proven to be critical for mitotic entry in medulloblastoma cells^40^ and for the platelet-derived growth factor (PDGF)-induced proliferation of vascular smooth muscle cells^41^. Since cell cycle arrest forms a major barrier for the induction of cellular pluripotency in somatic cells^42, 43^, our results together with previous discoveries suggest that the channel blocker-suppressed and ionophore-enhanced cell reprogramming in HDFs could be, at least partially, attributed to the influence of potassium permeability on the cell cycle of reprogrammed cells.

Since NANOG expression was affected by TEA and SAL in reprogrammed cells (Fig. 4D,E), we speculated that the expression of other endogenous genes critical for the establishment of cellular pluripotency may be perturbed during cell reprogramming with potassium modulators. As shown in Fig. 5D, the expression of endogenous *POU5F1, SOX2, KLF4* and *MYC* genes in reprogrammed HDFs was influenced by TEA and SAL treatment at the early stage of cell reprogramming. On the other hand, unreprogrammed HDFs with similar treatment appeared to show marginal changes in the expression of these endogenous genes (Figure S5). TEA significantly inhibited the expression of endogenous *POU5F1* and *SOX2* genes in cells reprogrammed with and without VPA (Fig. 5D, upper panel), providing another possible mechanism leading to the deficiency of hiPSC formation from reprogrammed cells with reduced potassium permeability. In contrast, SAL considerably enhanced the expression of endogenous *POU5F1* and *SOX2* genes in cells reprogrammed without VPA (Fig. 5D, lower panel), supporting our discovery that SAL assisted the generation of hiPSCs (Fig. 4C). Since POU5F1 and SOX2 play a critical role in counteracting lineage specification signaling and interact with other factors to modulate self-renewal and pluripotency in hPSCs^44–46^, our findings suggest that the decrease of intracellular potassium, as a result of increased potassium permeability in cells, could be important for the optimization of endogenous POU5F1 and SOX2 signaling and the efficient establishment of pluripotency in reprogrammed cells.

Unlike the SAL-enhanced expression of endogenous *POU5F1* and *SOX2* genes in HDFs reprogrammed without VPA, the expression of these two genes in the HDFs reprogrammed with VPA appeared to be unaffected by salinomycin treatment (Fig. 5D, lower panel). This paradoxical finding suggests that the suppressive effect of SAL on hiPSC formation during VPA-assisted reprogramming is presumably mediated by other molecular mechanisms. In addition, it reflects the complex molecular features that can be influenced by changing cell membrane permeability to potassium in reprogrammed cells.

**Figure 6:**
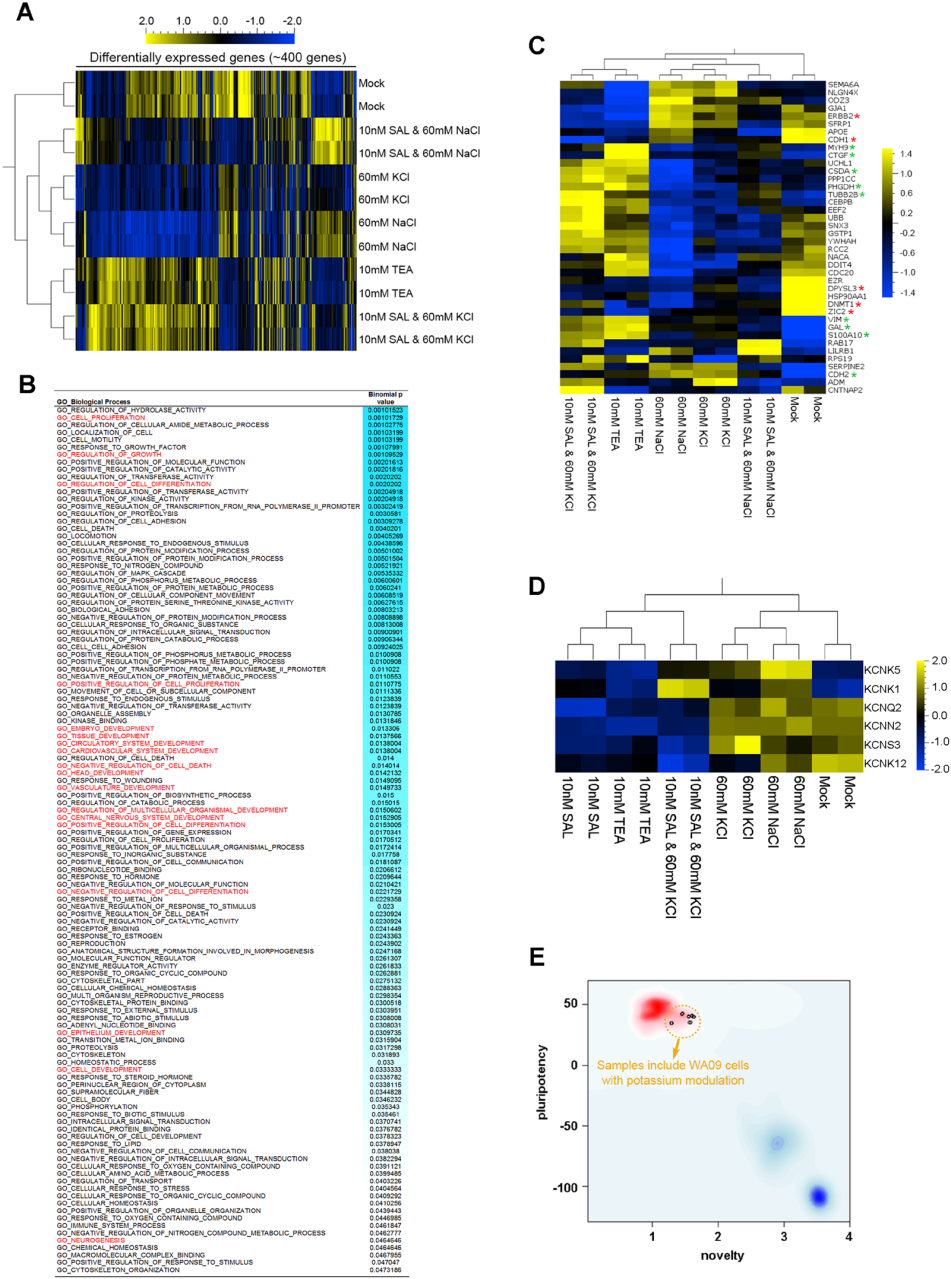
Global gene expression profiling and differentially expressed genes in control and potassium modulator-treated WA09 hESCs. WA09 hESCs with the indicated treatment for 48 hours were collected for RNA isolation and global gene expression profiling. (**A**) A heat map representation of the relative expression levels of ~420 differentially expressed genes in the WA09 hESCs with the indicated treatment. (**B**) Gene ontology analysis revealed that genes differentially expressed due to the modulation of intracellular potassium content in WA09 hESCs were highly enriched in biological processes including cell proliferation, cell differentiation, and developmental events (highlighted in red). (**C**) A heat map representation of the relative expression levels of a selected panel of genes highly relevant to embryogenesis, regulation of cell differentiation, development of the central nervous system, and development of the cardiovascular system. *Red asterisks:* genes known for being highly expressed in pluripotent cells. *Green asterisks:* genes associated with the differentiation and development of specific cell lineages. (**D**) A heat map representation of the relative expression levels of selected genes that encode potassium channel proteins. (**E**) The pluripotency of WA09 hESCs with the modulation of intracellular potassium contents was examined using the PluriTest^®^ based on global gene expression profiles. Localization of the cell samples subjected to mock, TEA (10 mM), SAL (10 nM), sodium chloride (60 mM), potassium chloride (60 mM), and combinatorial treatment at the upper-left corner of the plot within the red area indicates that these cells are pluripotent in general.

Through global gene expression profiling followed by differential expression analysis, we have identified a group of genes (~420 genes) that are significantly upregulated or downregulated at the transcriptional level in WA09 hESCs with the modulation of their potassium contents (Fig. 6A). Many of these genes are involved in the regulation of cell proliferation, cell growth, differentiation, development of specific cell lineages (Fig. 6B), indicated by gene ontology analysis. Among the selected genes that are associated with embryogenesis, regulation of cell differentiation, development of the central nervous system, and development of the cardiovascular system, many of these genes (*e.g., S100A10*, *CTGF*, *CSDA*, *PHGDH*, *TUBB2B*, *MYH9, VIM*, *CDH2*) known for their significance in the differentiation of specific cell lineages are upregulated in WA09 hESCs with treatment (TEA treatment or the concomitant treatment of SAL and 60 mM KCl) that retains potassium in the cells (Fig. 6C and Figure S6). In contrast, several genes (*e.g., ERBB2, CDH1, DNMT1 and ZIC2*) that are linked to cellular pluripotency are downregulated in the cells with such treatment (Fig. 6C and Figure S6). These findings further attest to the influence of potassium modulation on the regulation of pluripotency and differentiation at the transcriptional level in hPSCs. Similar transcriptional responses may also occur in somatic cells that receive potassium modulation during reprogramming, tuning the molecular threshold for successful establishment of cellular pluripotency. Although TEA and SAL may appear to downregulate the expression of genes that encode potassium channel proteins, undifferentiated hPSCs clearly express the targets (Fig. 6D) of potassium channel modulators used in our studies.

## Discussion

Although their contents in cells may be trace, many inorganic elements are essential for cell vitality and normal function. It has been known that several inorganic elements including Zn and Ca are critically involved in the activation of mammalian eggs and the development of blastocysts^47, 48^, highlighting a possible role of inorganic elements in the regulation of pluripotent cell formation and cellular pluripotency. In this study, we identified potassium as a pluripotency-associated inorganic element and demonstrated that the alteration of cell membrane permeability to potassium and the resulting change in intracellular potassium content affect the maintenance and establishment of cellular pluripotency in *bona fide* hPSCs and reprogrammed cells. These pleiotropic effects are likely mediated through the modulation of cell cycle and the perturbation of endogenous POU5F1 and SOX2 expression.

Due to the stochastic variations that potentially exist among different hPSC lines and differentiated derivatives, we measured the intracellular content of potassium in a panel of pluripotent and non-pluripotent cell samples, including a variety of hPSC lines, their isogenic differentiated derivatives and the primary cells used for reprogramming. Proven using two analytical platforms, the undifferentiated pluripotent cells derived from different origins and methods consistently showed a lower potassium content compared with their corresponding differentiated derivatives and primary cells. This finding supports that a reduced potassium content is a feature specifically associated with cellular pluripotency in human cells. Similar to other pluripotency-associated features that can show certain variations among different lines of hPSCs^10, 12^, the variation of absolute contents of potassium in different hPSCs was noticed in this study. This variation may be relevant to the differentiation propensities and/or functionalities of hPSCs and their differentiated derivatives, requiring a future study to test that. In contrast to the recent discovery that suggests the role of Zn in maintaining the cellular pluripotency of rodent embryonic stem cells^25^, the intracellular content of Zn did not appear to be correlated with the cellular pluripotency of human cells in our study. Although this discrepancy may be due to the species difference and require additional tests to be validated, the results of our and the preceding studies indicate that multiple inorganic elements could contribute to the regulation of cellular pluripotency.

Using pharmacological tools including potassium channel modulators and a potassium ionophore, we demonstrated that intracellular potassium content is not only a pluripotency-associated feature but also a critical factor that functionally impacts hPSCs. While the reduction of cell membrane permeability to potassium downregulated the core components of pluripotency signaling in hPSCs, the increase of the permeability enhanced the signaling. Similar effects of altering the potassium permeability on cellular pluripotency signaling were also found in human somatic cells undergoing acquisition of cellular pluripotency. In addition to our findings (Fig. 6D), the expression of functional potassium channels that are sensitive to two structurally distinct, small-molecule blockers (TEA and 4-AP) used in our study was previously observed in hPSCs^37, 38^. Compared with mesenchymal stem cells that are not pluripotent, hPSCs appeared to express more gene transcripts for these potassium channel proteins^37^. These findings suggest that the membrane permeability to potassium in undifferentiated hPSCs is intrinsically programmed at the gene expression level, in order to favor a low intracellular potassium content and sustain pluripotency signaling.

We also found that apoptosis can be preferentially induced by 4-AP in hPSCs rather than their isogenic non-pluripotent cells, highlighting the high sensitivity of hPSCs to the potassium channel blocker and the potential application of potassium channel inhibition in regenerative medicine. For cell therapy, residual undifferentiated hPSCs in differentiated derivatives are potentially tumorigenic and need to be removed for the safety of therapy recipients^49^. In addition, use of impure differentiated derivatives that contain undifferentiated hPSCs for research purposes may bias evaluations of drug efficacy and toxicity, differentiation potential, or other cellular responses. Therefore, selection mechanisms for eliminating pluripotent cells from differentiated cells are essential, not only for the purification of differentiated hPSCs intended for clinical use but also for the quality control of cell samples in basic research. A few approaches that target pluripotency-associated cell surface antigens^12, 50–55^ and metabolic stress^56–58^ have been established for the cell purification purpose. Since 4-AP has been used in clinical settings^59^, with further optimization, the selective toxicity of 4-AP in hPSCs may be developed into another simple, effective and economical method for the removal of pluripotent cells without damaging functional cells.

Many factors that have intricate crosstalk were expected to be influenced by the modulation of intracellular potassium. Although challenging, we sought to determine what mechanisms may underlie the effects of intracellular potassium alteration on cellular pluripotency in hPSCs and reprogrammed cells. Consistent with previous findings, we showed that the progression of cell cycle in somatic cells used for cell reprogramming was hindered by the reduction, but facilitated by the enhancement of cell membrane permeability to potassium. The G1 phase of cell cycle in HDFs appears to be predominantly affected by the modulation of cell membrane permeability to potassium (Fig. 5A). It is possible that the alteration of intracellular potassium may perturb the function of the G1/S checkpoint in the cells. Although it is not within the scope of this study, it would be scientifically interesting in the future to examine the molecular components that are associated with cell cycle regulation and influenced by the modulation of intracellular potassium in the context of cell reprogramming.

In addition to the cell cycle, the induction of endogenous gene expression that is pivotal for the pluripotency signaling network was significantly altered due to the change of cell membrane permeability to potassium at the early stage of reprogramming. It has been reported that the DNA binding activity of transcription factors including Tp53, Forkhead, HIF, Pou2f1 and NF-κB in fetal rat cortical neurons can be affected by the level of intracellular potassium, subsequently triggering the expression alterations of certain downstream genes that contain the responsive elements of these transcription factors^60, 61^. Since many core components of pluripotency signaling networks are transcription factors that can form reciprocal regulatory loops^62^, their transcriptional activity may be drastically changed by the alteration of intracellular potassium content, undermining or strengthening the pluripotency signaling in a context-dependent manner. Our study of gene expression profiles at a global level in hPSCs with altered potassium contents provides additional information for probing how potassium regulates pluripotency signaling. Although cellular pluripotency may not be rapidly lost in hPSCs in response to treatment (TEA treatment or the concomitant treatment of SAL and 60 mM KCl) that retains intracellular potassium (Fig. 6E), it is clear that the alterations of cell membrane permeability to potassium in hPSCs can impact the expression of many pluripotency-associated and differentiation-associated factors (Fig. 6B and C) in addition to the endogenous *POU5F1*, *SOX2*, *KLF4* and *MYC* genes. These findings support that the manipulation of intracellular potassium contents is indeed a feasible strategy for priming human cells for differentiation or reprogramming by tipping the balance of these gene expression networks.

While we primarily focused on examining the cell cycle and gene expression alterations caused by the perturbation of intracellular potassium in this study, it is important to remember that other mechanisms such as epigenetic and post-transcriptional regulations may also be affected by intracellular potassium change during the establishment and maintenance of cellular pluripotency. Interestingly, a recent study provided clear evidence in support of that calcium-activated calcineurin signaling plays a biphasic role in somatic cell reprogramming through the modulation of cell cycle progression and histone methylation at the *Sox2* and *Klf4* gene promoters^63^. Since calcium influx can be induced by cell membrane hyperpolarization^64, 65^, SAL-induced decrease of intracellular potassium may trigger hyperpolarization, calcium influx, and subsequently the calcineurin signaling in reprogrammed fibroblasts and hPSCs. However, in testing the expression and localization of calcineurin A in response to the 48-hour treatment of SAL in hiPSCs, we found no obvious difference (Figure S7). In addition, using calcium imaging techniques, we analyzed their intracellular calcium levels before and after TEA and SAL treatment but barely detected any calcium flux induced by TEA and SAL in hiPSCs (Figure S7). These findings suggest that cellular pluripotency changes induced by potassium perturbation in human cells appear to be independent of calcium signaling. Nevertheless, exploring the effect of intracellualr potassium change on other regulatory mechanisms (*e.g*., epigenetic modulation and mesenchymal-epithelial transition) in future studies is still necessary.

In summary, the alterations of inorganic elements in cells are potentially an indication and a driving force for distinct cellular states. We reason that the intracellular level of potassium may be associated with the maintenance and establishment of cellular pluripotency in human cells. Using two analytical approaches for profiling inorganic components in cell samples, we identify potassium as a pluripotency-associated element for the first time. Compared with non-pluripotent cells, undifferentiated hPSCs typically contain less potassium. Changes in intracellular potassium due to altered membrane permeability perturb the core components of pluripotency signaling and cell reprogramming. Our work also provides mechanistic and application insights into how using pharmacological tools to manipulate intracellular potassium would be an effective and useful strategy to control cellular pluripotency in human cells. We expect that the modulation of a pluripotency-associated inorganic element like potassium will make a novel and useful strategy for harnessing hPSCs for research and regenerative medicine in the future.

## Methods

### Cell culture

HDF51 (HDF-f; ScienCell Research Laboratories, Carlsbad, CA) cells, and CF-1 mouse embryonic fibroblasts (MEF) (ATCC, Manassas, VA) were cultured in DMEM containing 10% fetal bovine serum (FBS; Thermo Fisher Scientific, Carlsbad, CA) at 37 °C. HM (HEMl; ScienCell Research Laboratories, Carlsbad, CA) cells were cultured in melanocyte medium (MelM; ScienCell Research Laboratories, Carlsbad, CA). WA09 hESCs were obtained from the WiCell Stem Cell Bank (WiCell Research Institute, Madison, WI). HDF51i-509, HMi-506 and PBMC418i-1506 hiPSCs were kindly provided by Dr. Jeanne F. Loring at the Scripps Research Institute. The information of cells used in this study was summarized in Table S1. The experiments using hPSCs were performed in compliance with the guidelines and approval of the institutional biosafety committee at UNTHSC. All cells were tested using the MycoAlert mycoplasma detection kit (Lonza, Walkersville, MD) and free of mycoplasma. For culturing undifferentiated hPSCs, we followed the previously described method^12^ for culturing hPSCs in the feeder cell-free condition, except the use of TeSR-E8 medium (Stemcell Technologies, Vancouver, Canada) and L7 hPSC passaging solution (Lonza, Walkersville, MD) in this study.

### Cell differentiation and reprogramming

For non-directed differentiation of hPSCs by embryoid body (EB) formation, hPSCs grown in the feeder cell-free condition were harvested using Accutase cell dissociation agent, pelleted and resuspended in the culture medium containing 10 µM Y27632 (Stemgent, Cambridge, MA). The cell suspension was placed into AggreWell plates (Stemcell Technologies, Vancouver, Canada) for 24 hours to form hPSC aggregates. The cell aggregates were collected with minimal trituration into bFGF-deficient DMEM/F12 medium with L-glutamine containing 20% KnockOut Serum Replacement, 100 µM non-essential amino acids, and 100 µM ß-mercaptoethanol (hESC medium; all components from Thermo Fisher Scientific, Carlsbad, CA) and plated into Ultra-low attachment multiwell plates (Corning, Corning, NY) for continuously cultured in suspension for 7 days with medium changing on the fourth day. At the end of suspension culture, EBs developed from the cell aggregates were plated into culture plates with gelatin coating and cultured in DMEM/F12 medium containing 15% FBS for an additional 7 days prior the following analysis. The protocol used to generate melanocytic differentiated derivatives of hPSCs was reported in a previous study^66^. To generate induced pluripotency in HDFs, we used retroviral vectors that have been used in a previous study^12^ to deliver four reprogramming factors (*POU5F1*, *SOX2*, *KLF4*, and *MYC* genes) into HDF51 cells. At a density of 1.5 × 10^4^ cells per well of a six-well plate, the transduced cells were seeded into six-well plates containing radiation-inactivated MEF feeder cells and cultured in hESC medium containing 12 ng/ml FGF2 (Biopioneer, San Diego, CA) for ~21 days with medium changing every other day. Tetraethylammonium and 4-aminopyridine were purchased from Sigma-Aldrich (St. Louis, MO). Salinomycin and diazoxide were purchased from Cayman Chemical (Ann Arbor, Michigan). For cell reprogramming to test the effects of the potassium channel modulators and ionophore on the reprogramming efficiency, tetraethylammonium, 4-aminopyridine, diazoxide and salinomycin were supplied in the culture medium containing 0 and 0.5 mM VPA (Stemgent, Cambridge, MA) for initial 8 days after the transduced cells split onto the feeder cells. The efficiency of reprogrammed cells to form hiPSC colonies was evaluated using the alkaline phosphatase staining kit II (Stemgent, Cambridge, MA). For cell reprogramming to test the effects of the potassium channel modulators and ionophore on the molecular level, the transduced cells were placed into matrigel (Corning, Corning, NY)-coated wells at a density of ~3.5 × 10^5^ cells per well of a six-well plate and cultured in MEF-conditioned medium supplied with tetraethylammonium, 4-aminopyridine, diazoxide and salinomycin for the indicated periods.

### Analysis of inorganic elements

Cells (4 × 10^5^) from each of different cell types were collected, quickly rinsed with normal saline and D-mannitol solution (308 mOsmol/L in deionized water), and pelleted. Each cell pellet was digested in 0.5 ml of 70% nitric acid, dried at 70 °C, and then dissolved in 0.7 ml of 2% nitric acid. In average, each microliter of the sample solution contained the inorganic content of ~571 cells. The aliquots of sample solutions were analyzed using the TXRF and ICP-MS techniques to determine the amount of inorganic elements in the same number of cells across different cell pellets. For TXRF analysis, 35 µl sample solution was dried on a polished quartz disc. The excitation of inorganic elements and the measurement of X-ray fluorescence photon emission of each element on the disc was performed using a S2 Picofox TXRF spectrometer (Bruker, Billerica, MA) equipped with a 40 W molybdenum alloy anode X-ray tube and a silicon drift detector with energy resolution of <150 eV at 100 kcps (Mn Kα). The measurement setting was 50 kV and 600 µA for an acquisition time of 300 seconds. Each disc was scanned four times with a disc rotation of 90 degrees between each scanning to obtain the average amount of X-ray fluorescence emitted by specific elements on the disc. The type and amount of each detected element in samples were called using the Picofox software for data acquisition and analysis (Bruker, Billerica, MA). For ICP-MS analysis, 500 µl sample solution was added to 1.5 ml of 2% nitric acid to form a diluted solution. One milliliter of each diluted solution was analyzed using a NexION 300D ICP-MS spectrometer (Perkin Elmer, Waltham, MA). Germanium and scandium (both from High-Purity Standards, Charleston, SC) were used as internal standards. A quality control standard solution containing 27 elements (High-Purity Standards, Charleston, SC) was diluted into various concentrations with 2% nitric acid and used to generate the standard curve for calculating the content of each element in sample solutions. The isotopes of elements with minimal interference were chosen for measurement. The setting of instrument parameters used for analysis was summarized in Table S2. To compare the elemental profiles from undifferentiated and differentiated WA09 hESCs across two analytical platforms with different detection limitations, the amount of each element was normalized to the total amount of 10 selected elements determined by the TXRF and ICP-MS techniques in every sample to calculate the ratio of each element in all 10 elements that we analyzed.

### Fluorescence staining

The general procedure for antibody-mediated fluorescence staining was previously described^12^. For the staining of pluripotency biomarkers in hPSCs and their differentiated derivatives, cells were plated into 24-well plates, fixed and permeabilized and incubated with primary antibodies against specific pluripotency biomarkers and fluorophore-conjugated secondary antibodies (Thermo Fisher Scientific, Carlsbad, CA). The primary antibodies used in the study were purchased from Cell Signaling Technology (POU5F1, cat# 2840; Calcineurin A/PPP3CA, cat# 2614) and EMD Millipore (NANOG, cat# MABD24). For the staining of intracellular potassium using a fluorescence indicator, cells were incubated with culture media containing 2 µM APG2-AM (TEFLabs, Austin, TX) for 1 hour. For labeling apoptotic cells, Alexa Fluor 488-conjugated annexin V (Thermo Fisher Scientific, Carlsbad, CA) were diluted in culture media according to the manufacturer’s instruction and reacted with cells at 37 °C in the dark for 15 minutes.

### Immunoblotting

The general procedure for immunoblotting was described in a previously published report^67^, except that cell lysates were prepared using M-PER mammalian protein extraction reagent (Thermo Fisher Scientific, Carlsbad, CA) containing protease inhibitor and phosphatase inhibitor cocktails (EMD Millipore, Billerica, MA). The primary antibodies used in this study were purchased from Cell Signaling Technology (POU5F1; cat# 2840), EMD Millipore (NANOG; cat# MABD24) and MP Biomedicals (ACTIN; cat# 08691001). HRP-conjugated secondary antibodies were from Jackson ImmunoResearch Laboratories (West Grove, PA). The antibodies used in this study have been used or validated in separate work previously published by our and other groups.

### Flow cytometry

For measuring the expression of NANOG in the reprogrammed cells, the cells were collected on the day 14 after they were split into plates containing the feeder cells. The collected cells were fixed using PBS containing 4% paraformaldehyde, perforated using PBS containing 0.1% Triton X-100, and reacted with Alexa Fluor 488-conjugated antibody targeting NANOG (cat# MABD24A4; EMD Millipore, Billerica, MA) at 4 °C for 25 minutes. The count of cells with fluorescence signal in each stained sample was determined using a FC500 flow cytometer equipped with CXP software (Beckman Coulter, Indianapolis, IN). For measuring the fluorescence intensity of APG2, cells were collected and analyzed on the flow cytometer immediately after APG2-AM staining. For the cell cycle analysis, ~2.5 × 10^5^ cells with drug treatment for the indicated period in each sample were harvested, washed with PBS, fixed in 40% ethanol in PBS at 4 °C for 24 hours, and stained in PBS containing 5 µg/ml propidium iodide (Thermo Fisher Scientific, Carlsbad, CA) and 50 µg/ml RNase A (Thermo Fisher Scientific, Carlsbad, CA) for 30 minutes. The DNA contents of ~2 × 10^4^ individual cells from each sample were analyzed using the FC 500 flow cytometer. The percentages of cells in each cell cycle phase were determined using the cell cycle analysis module of the FlowJo v10 software. For quantifying the percentages of apoptotic cells in cell samples, samples (~1 × 10^6^ cells per sample) stained with Annexin V-Alexa Fluor 555 (Thermo Fisher Scientific, Carlsbad, CA) according to the manufacturer’s instruction were analyzed using a SH800Z cell sorter (Sony Biotechnology, San Jose, CA).

### Calcium imaging

Cells grown in a recording chamber and preloaded with the calcium indicator Fura 2-AM (Thermo Fisher Scientific, Carlsbad, CA) in culture medium for 45 minutes were rinsed three times with calcium-containing Hank’s buffered salt solution (HBSS) before the recording of Fura 2 fluorescence changes in the cells. The cells were challenged using salinomycin and ionomycin (Sigma-Aldrich, St. Louis, MO). The intensity of intracellular Fura 2 fluorescence was acquired and analyzed using an Olympus IX70 fluorescence microscope (Olympus, Center Valley, PA) equipped with MetaFluor fluorescence ratio imaging software (Molecular Devices, Sunnyvale, CA).

### Gene expression analysis by qRT-PCR and microarrays

Total RNA was isolated from cell samples using the mirVana miRNA Isolation Kit (Thermo Fisher Scientific, Carlsbad, CA). The iScript Reverse Transcription Supermix (Bio-Rad, Hercules, CA) was used to generate the cDNA of total RNA samples. To measure the expression of endogenous *POU5F1*, *SOX2*, *KLF4* and *MYC* genes in cell samples, qRT-PCR was performed using the cDNA samples, iTaq Universal SYBR Green Supermix (Bio-Rad, Hercules, CA) and specific primers that target untranslated regions of four endogenous gene transcripts (absent in exogenously expressed reprogramming factors)^10^. The expression of *ACTB* gene detected by a pair of specific primers (^5′^GGCGGCACCACCATGTACCCT^3′^ and ^5′^AGGGGCCGGACTCGTCATACT^3′^) was used as the internal control. Global gene expression profiling was performed using HT-12v4 Human Gene Expression BeadChips (Illumina, Hayward, CA), according to the manufacturer’s instructions. The gene expression array data have been deposited with links to an accession number GSE90826 in the Gene Expression Omnibus (GEO). Data were filtered for detection *P* value < 0.01 in GenomeStudio (Illumina, Hayward, CA), and normalized using the LUMI package with RSN (Robust spline normalization) algorithm in R. Qlucore Omics Explorer 3.0 was used to perform differential gene expression analysis, hierarchical clustering, and the ontology analysis of differentially expressed genes. The test of cellular pluripotency based on the transcriptomic features of cell samples was performed using the PluriTest^3^. Multiplex qRT-PCR was performed using Taqman^®^ assays for the *DNMT1*, *CDH1*, *VIM, MYH9* and *ACTB* (internal control) genes (cat# Hs00945875_m1, Hs01023895_m1, Hs00958111_m1, Hs00159522_m1 and Hs03023943_g1; Thermo Fisher Scientific, Carlsbad, CA), according to the manufacturer’s instructions.

### Statistical analysis

Data reported in this work were reproducible in three biological replicates and presented as mean ± standard deviation. The significance of differences in comparisons was determined by the two-tailed Student’s *t*-test. Values of *P* < 0.05 were considered statistically significant.

## Electronic supplementary material

Supplementary Information
