# Supplementary material for: Potassium as a pluripotency-associated element identified through inorganic element profiling in human pluripotent stem cells
Source: Sci Rep. 2017 Jul 10;7:5005. doi: 10.1038/s41598-017-05117-2 (PMC5504050; doi:10.1038/s41598-017-05117-2)

Supplementary Information

Potassium as a pluripotency-associated element identified through inorganic element profiling in human pluripotent stem cells

Victor J.T. Lin[1], Ashwini Zolekar[1], Yi Shi[1,*], Bhuvaneswari Koneru[1], Slobodan Dimitrijevich[2,3], Anthony J. Di Pasqua[1,*], Yu-Chieh Wang[1,¶]

[1] Department of Pharmaceutical Sciences, University of North Texas Health Science Center, Fort Worth, TX, USA

[2] Cardiovascular Research Institute, University of North Texas Health Science Center, Fort Worth, TX, USA

[3] UHV Technologies, Inc., Fort Worth, TX, USA

* Current address: Department of Pharmaceutical Sciences, School of Pharmacy, Binghamton University, NY, USA

[¶] Correspondence should be addressed to:

Yu-Chieh Wang, Ph.D.
Department of Pharmaceutical Sciences
The University of North Texas Health Science Center
3500 Camp Bowie Boulevard, RES-314G
Fort Worth, Texas 76107
Tel: (817) 735 2944
Fax: (817) 735 2603
Email: yu-chieh.wang@unthsc.edu

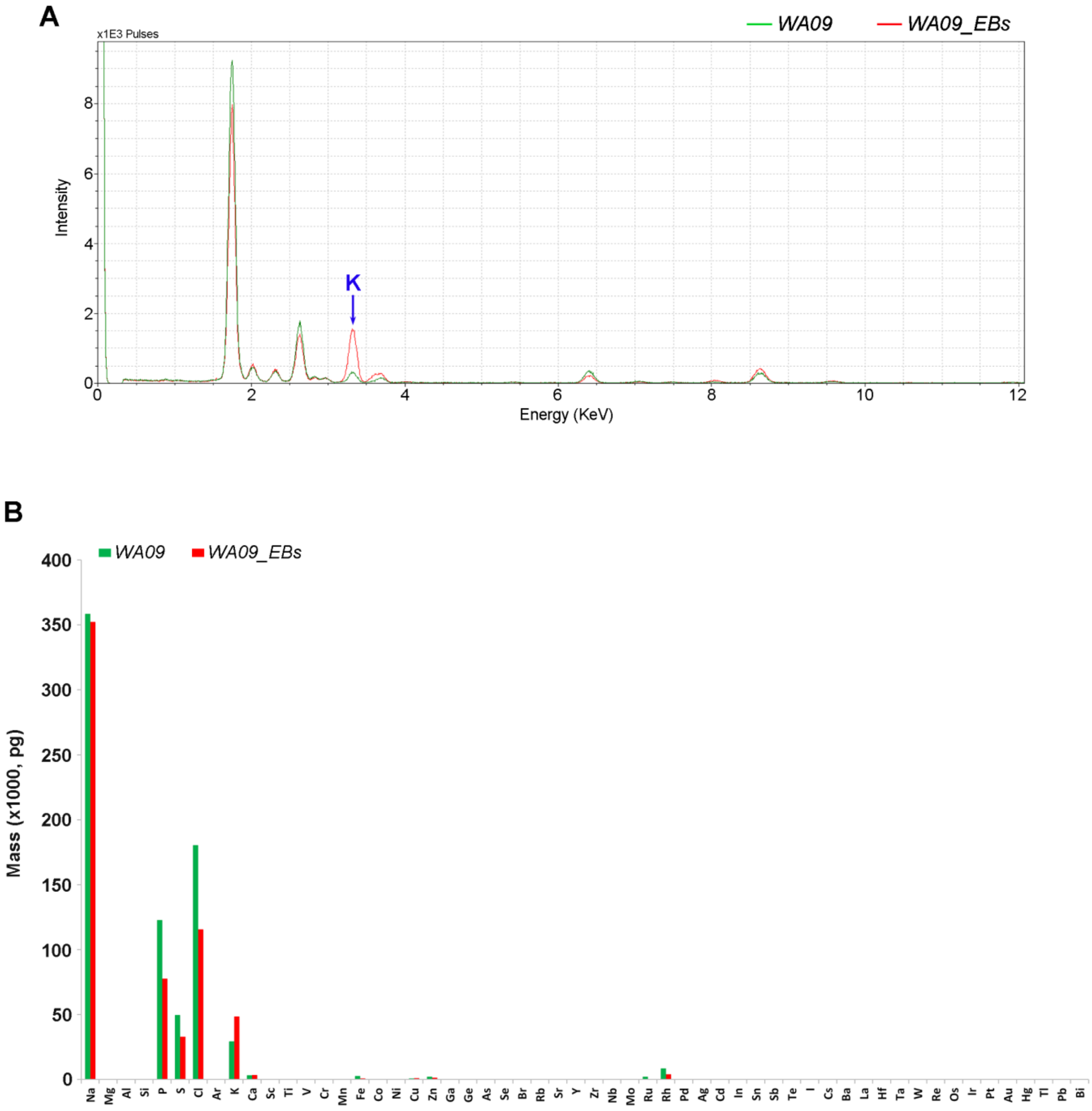

Figure S1, related to Figure 1. Profiling of inorganic elements in hESCs and their differentiated derivatives using TXRF analysis. (**A**) The histogram representation of X-ray fluorescence spectra obtained from the samples of WA09 hESCs and their EBs (WA09_EBs). (**B**) The amounts of 56 inorganic elements on two sample discs were determined according to the spectra and intensity of emitted X-ray fluorescence from a single scanning.

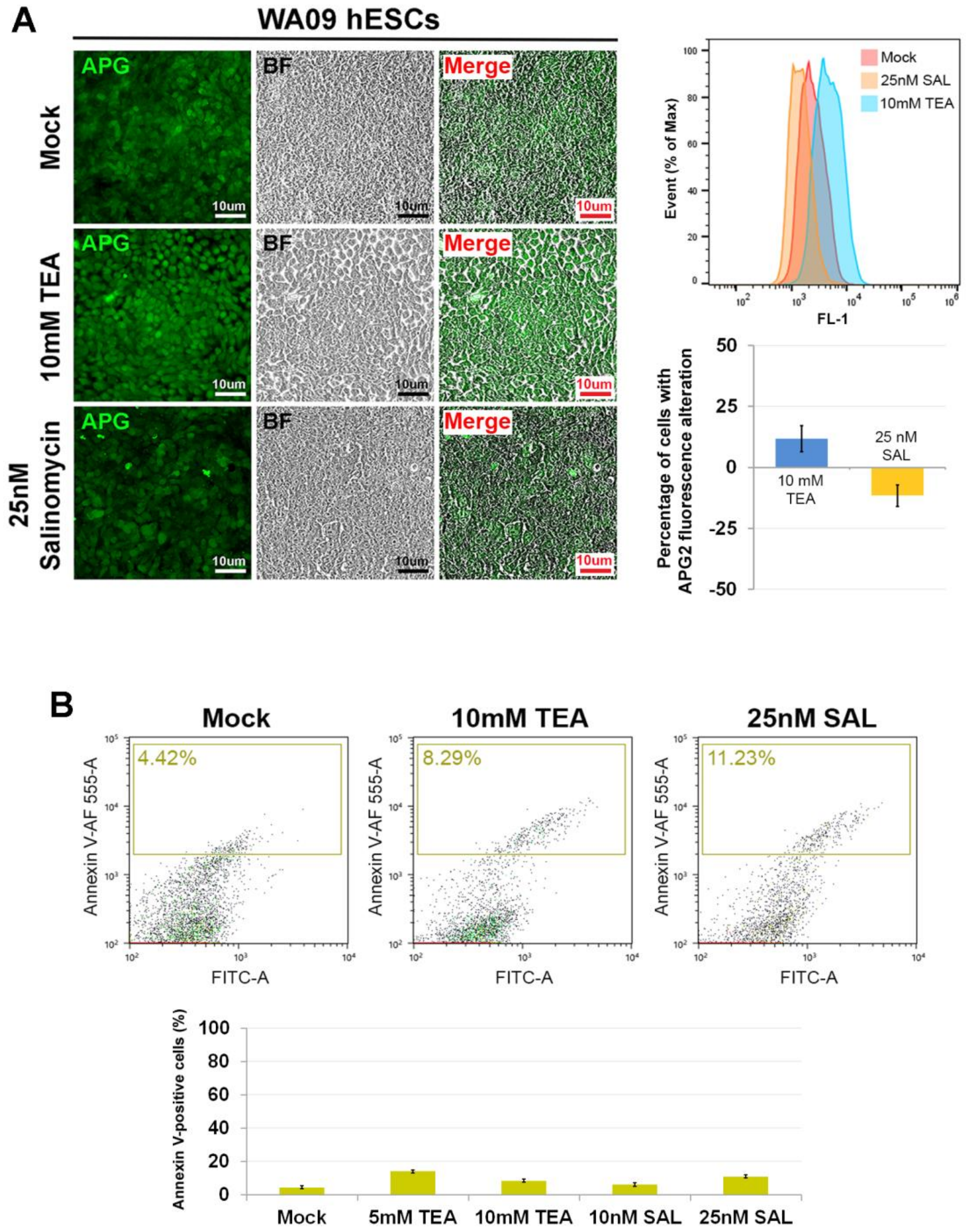

Figure S2, related to Figure 1 and Figure 2. APG2-AM and Annexin V staining of WA09 hESCs treated with 10 mM tetraethylammonium (TEA) and 25 nM salinomycin (SAL). (**A**) Compared with WA09 hESCs that received mock treatment, the cells with the treatment of 10 mM TEA showed enhanced APG2 fluorescence. In contrast, the treatment of 25 nM salinomycin led to the reduction of APG2 fluorescence in the cells. *Left panel:* Fluorescence microscopy imaging of the cells. Cells were treated for 72 hours. *APG:* APG2-AM staining. *BF:* Bright field. *Upper-right panel*: Histogram representation of flow cytometry analysis in the cells. *Lower-right panel:* The percentages of cells with APG2 fluorescence alteration in TEA- and SAL-treated cells, compared with control cells. Cells received the treatment of TEA and SAL for 1 hour prior to collection for the analysis. The positive number indicates the percentage of the treated cells with increased APG2 fluorescence; the negative number indicates the percentage of the treated cells with decreased APG2 fluorescence. (**B**) The treatment of 5 and 10 mM TEA and 10 and 25 nM SAL for 72 hours induced a very limited increase of apoptosis in WA09 hESCs. *Upper panel:* Dot plot representation of flow cytometry analysis in the cells. *Lower panel:* The percentages of Annexin V-positive cells in TEA- and SAL-treated cells. All data in each bar graph are presented as mean ± standard deviation (n=3).

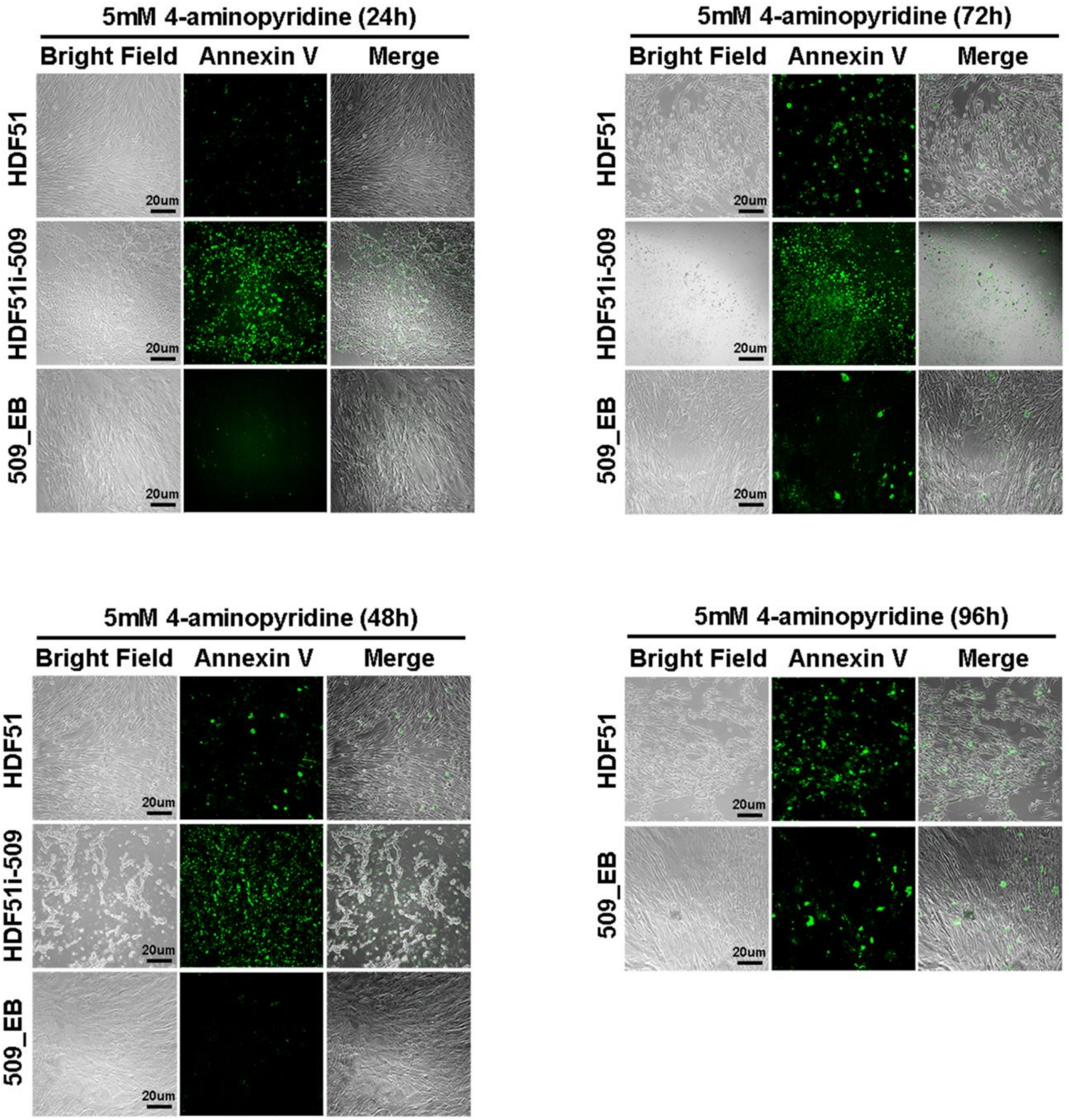

Figure S3, related to Figure 3. Potassium channel blocker 4-aminopyridine preferentially induced apoptosis in undifferentiated hPSCs at 5mM in a time-dependent manner. Staining of Alexa Fluor 488-conjugated annexin V in hiPSCs (HDF51i-509), their differentiated derivatives (509_EB) and somatic cells used for reprogramming (HDF51) with the treatment of 5 mM 4-aminopyridine indicated that most hiPSCs were eliminated by the treatment within 72 hours due to apoptosis. In contrast, non-pluripotent cells (HDF51 and 509_EB) showed limited cytotoxicity in response to the treatment.

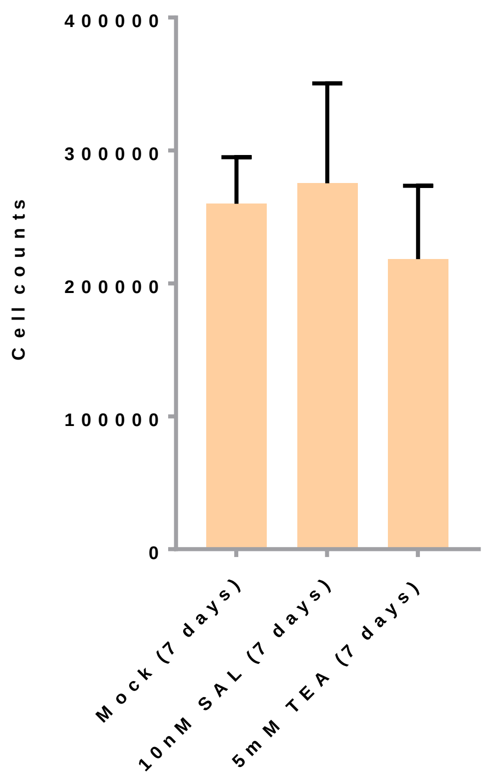

Figure S4, related to Figure 4. The cell counts of CF-1 MEFs in response to the 7-day treatment of tetraethylammonium (TEA) and salinomycin (SAL). The numbers of $1x10^4$ MEFs that received the mock and drug treatment for 7 days were determined by manual counting. No significant inhibition of growth was observed in the cells treated with TEA and SAL. All data are presented as mean ± standard deviation (*n*=3).

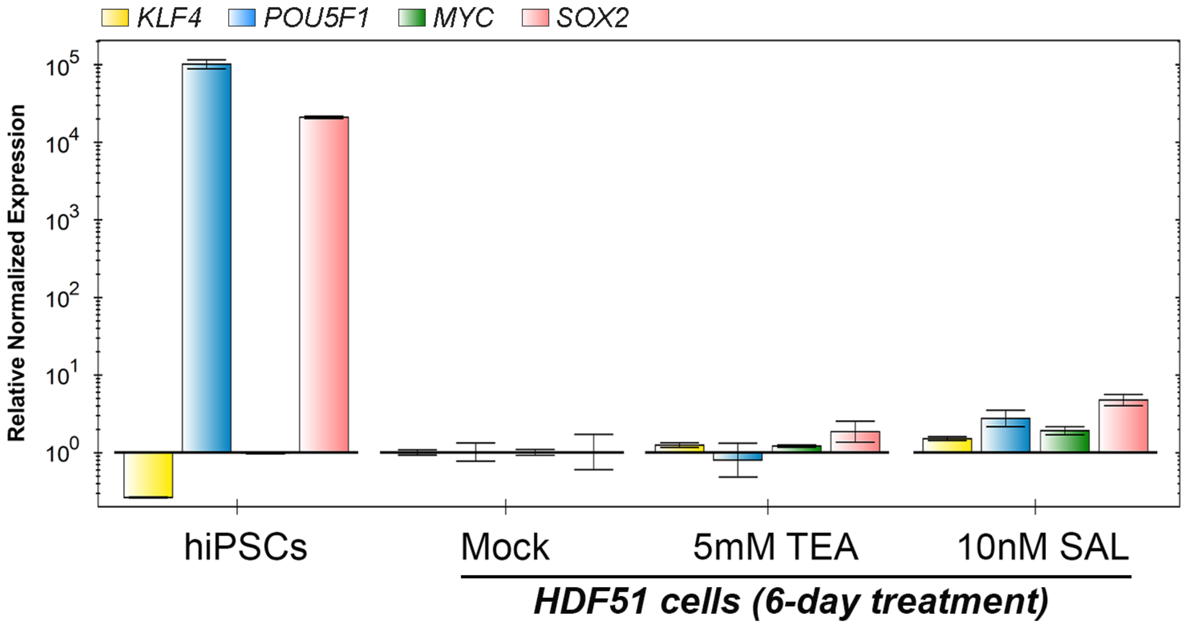

Figure S5, related to Figure 5. The expression of the endogenous *POU5F1*, *SOX2*, *KLF4* and *MYC* genes in HDFs with the indicated treatment of TEA and SAL for 6 days was analyzed by qRT-PCR. All data are presented as mean ± standard deviation (*n*=3).

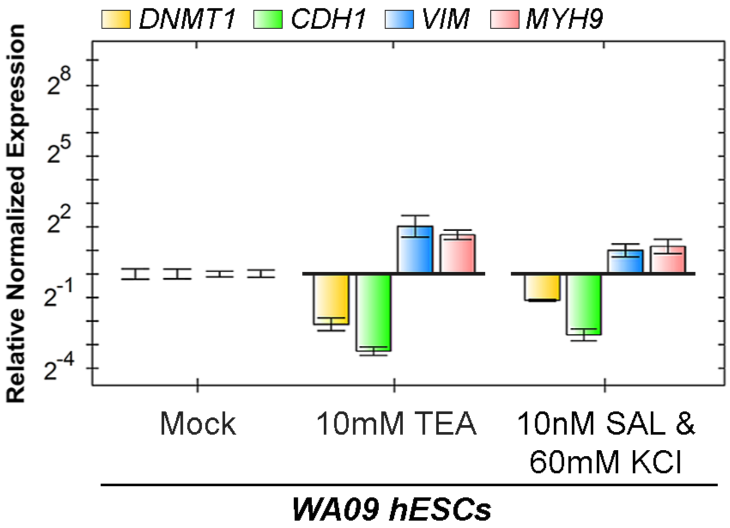

Figure S6, related to Figure 6. The expression of the selected pluripotency-associated (*DNMT1* and *CDH1*) and differentiation-associated (*VIM* and *MYH9*) genes in WA09 hESCs with the indicated treatment for 48 hours was analyzed by qRT-PCR. These genes were among the differentially expressed genes identified from global gene expression profiling. All data are presented as mean ± standard deviation (n=3).

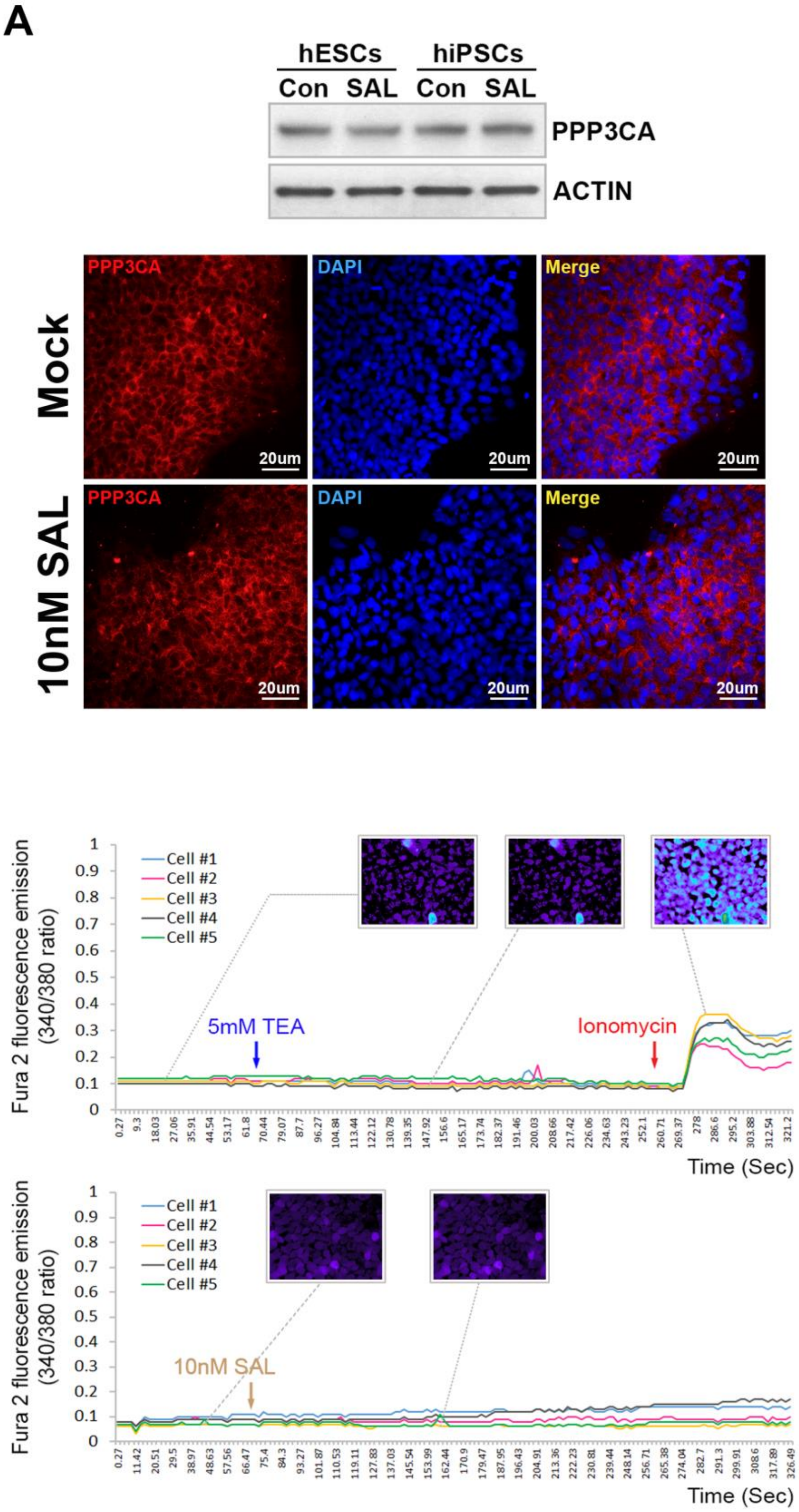

Figure S7, related to Figure 6. Analysis of calcium signaling in hPSCs with potassium perturbation. (**A**) *Upper panel:* The expression of calcineurin A α subunit (PPP3CA) in WA09 hESCs and HDF51i-509 hiPSCs that received 10nM salinomycin (SAL) for 48 hours. No obvious difference in PPP3CA expression was found. *Lower panel:* The localization of PPP3CA showed no obvious change with the indicated treatment in HDF51i-509 hiPSCs. (**B**) Unlike ionomycin treatment, the treatment of tetraethylammonium (TEA) and SAL did not trigger definitive calcium flux in HDF51i-509 hiPSCs. The insets are microscopic images of Fura 2 fluorescence in cells photographed at the indicated time points.

**Table S1. The list of cultured cells used in the study**		
**Sample Name**	**Registry Name**[a]	**Note**[b]
**Human embryonic stem cells**		
WA09	WA09	Feeder cell-free culture on Matrigel, passaged using L7 hPSC passaging solution
**Induced pluripotent stem cells from Human Dermal Fibroblasts (HDF)**		
HDF51i-509[c]	TSRI51i-HDF509	Sendai virus-mediated reprogramming in HDF51 cells; Feeder cell-free culture on Matrigel, passaged using L7 hPSC passaging solution
HDF51i-2501	NA	Self-replicative RNA-mediated reprogramming in HDF51 cells; Feeder cell-free culture on Matrigel, passaged using L7 hPSC passaging solution
**Induced pluripotent stem cells from Human Epidermal Melanocytes (HEM)**		
HMi-506[c]	TSRIi-HEMl506	Sendai virus-mediated reprogramming in HM cells; Feeder cell-free culture on Matrigel, passaged using L7 hPSC passaging solution
**Induced pluripotent stem cells from Human Peripheral Mononuclear Cells (PBMC)**		
PBMC418i-1506[c]	NA	Episomal vector-mediated reprogramming in human peripheral mononuclear cells; Feeder cell-free culture on Matrigel, passaged using L7 hPSC passaging solution
**Cells used for reprogramming**		
HDF51 (HDF-f)[d]	NA	Human dermal fibroblasts, fetal skin; purchased from Sciencell
HM (HEMl)[d]	NA	Human epidermal melanocytes, neonatal skin; purchased from Sciencell

a. Name of cell line submitted to University of Massachusetts (UMass) International Stem Cell Registry
b. Somatic cell type, reprogramming method, culture condition, source of cells
c. Cells kindly provided by Dr. Jeanne F. Loring at the Scripps Research Institute
d. Nomenclature used by the vendor

Table S2. The setting of instrument parameters for ICP-MS analysis (Related to Figure 1)

Parameter	Setting
ICP radio frequency generator power	1600
Plasma Gas Flow (L min-1)	18
Auxiliary Gas Flow (L min-1)	1.2
Nebulizer Gas Flow (L min-1)	1.02
Collision Gas (He) Flow (L min-1)	4.4-4.7
Sample Cones	Nickel/Aluminum
Modes	Standard (STD) and Kinetic Energy Discrimination (KED)
Dwell Time	50 ms
Internal Standards	Sc-45 (STD Mode; 20 ppb) and Ge-72 (KED Mode; 100 ppb)
Replicates	3
Time to Change Modes	60 s

The original Western blotting images of Figure 2A before cropping (4 quadrants indicate 4 sub-panels in Figure 2A)

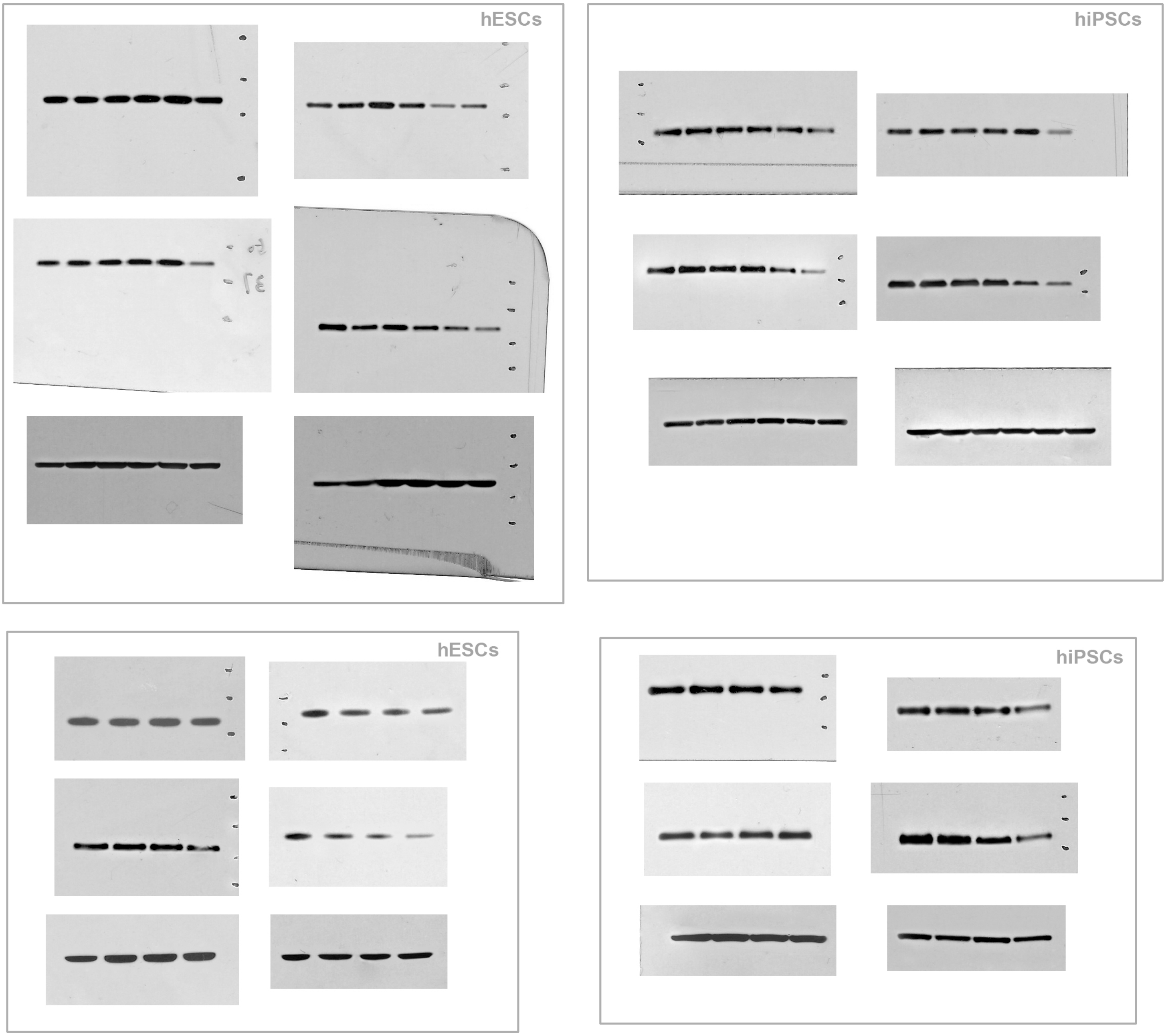

The original Western blotting images of Figure 2B before cropping (4 quadrants indicate 4 sub-panels in Figure 2B)

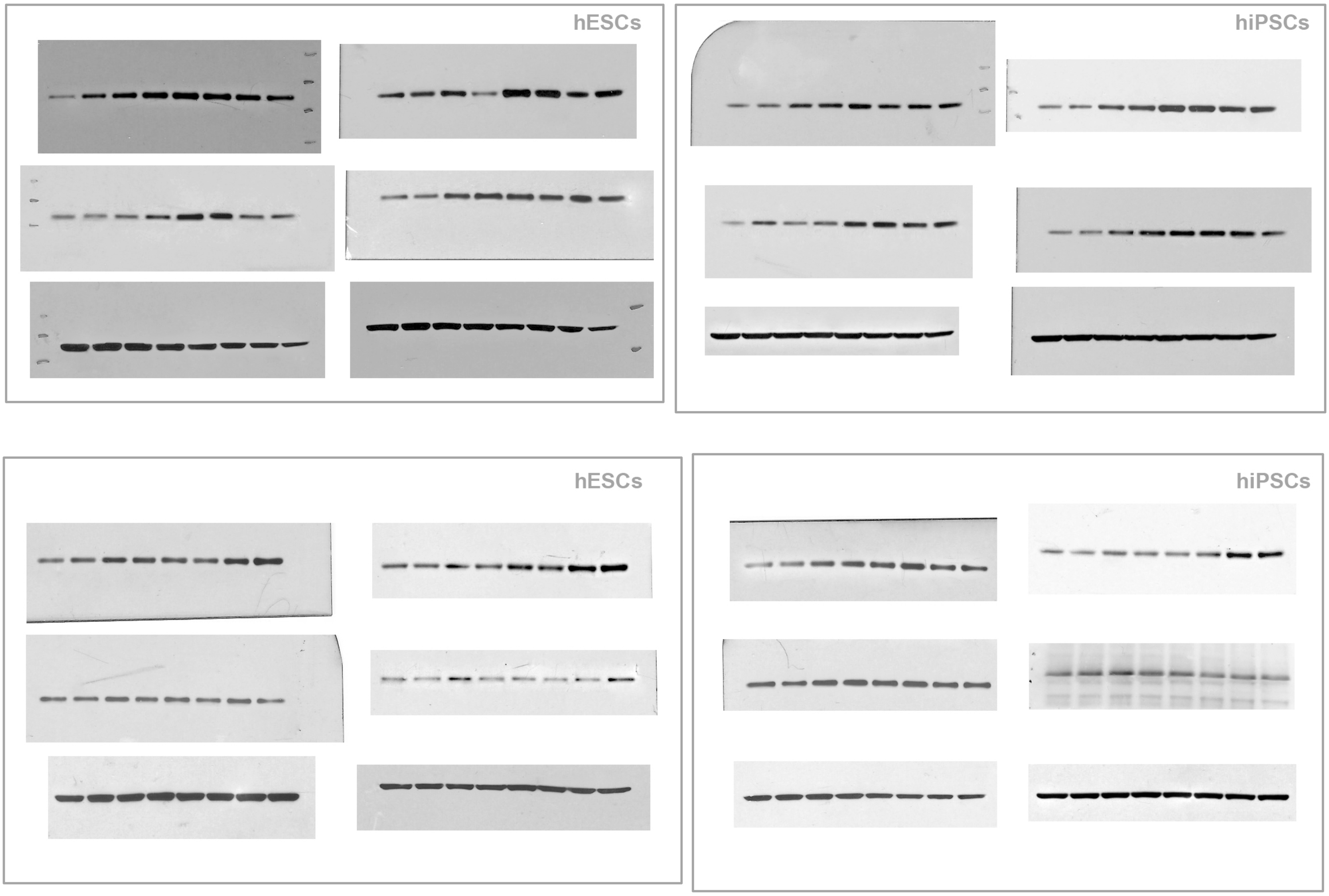

The original Western blotting images of Figure 2D before cropping

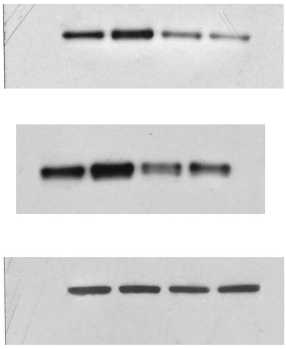

The original Western blotting images of Figure 2E before cropping

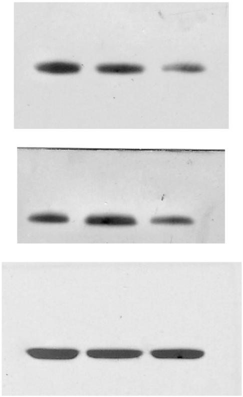

Supplement: Supplementary file 1 — Supplementary Information [file 41598_2017_5117_MOESM1_ESM.pdf]
